# Investigation of Polyurea-Crosslinked Silica Aerogels as a Neuronal Scaffold: A Pilot Study

**DOI:** 10.1371/journal.pone.0033242

**Published:** 2012-03-20

**Authors:** Firouzeh Sabri, Judith A. Cole, Michael C. Scarbrough, Nicholas Leventis

**Affiliations:** 1 Department of Physics, University of Memphis, Memphis, Tennessee, United States of America; 2 Department of Biological Sciences, University of Memphis, Memphis, Tennessee, United States of America; 3 Department of Biological Sciences, University of Memphis, Memphis, Tennessee, United States of America; 4 Department of Chemistry, Missouri University of Science and Technology, Rolla, Missouri, United States of America; RMIT University, Australia

## Abstract

**Background:**

Polymer crosslinked aerogels are an attractive class of materials for future implant applications particularly as a biomaterial for the support of nerve growth. The low density and nano-porous structure of this material combined with large surface area, high mechanical strength, and tunable surface properties, make aerogels materials with a high potential in aiding repair of injuries of the peripheral nervous system. However, the interaction of neurons with aerogels remains to be investigated.

**Methodology:**

In this work the attachment and growth of neurons on clear polyurea crosslinked silica aerogels (PCSA) coated with: poly-L-lysine, basement membrane extract (BME), and laminin1 was investigated by means of optical and scanning electron microscopy. After comparing the attachment and growth capability of neurons on these different coatings, laminin1 and BME were chosen for nerve cell attachment and growth on PCSA surfaces. The behavior of neurons on treated petri dish surfaces was used as the control and behavior of neurons on treated PCSA discs was compared against it.

**Conclusions/Significance:**

This study demonstrates that: 1) untreated PCSA surfaces do not support attachment and growth of nerve cells, 2) a thin application of laminin1 layer onto the PCSA discs adhered well to the PCSA surface while also supporting growth and differentiation of neurons as evidenced by the number of processes extended and b3-tubulin expression, 3) three dimensional porous structure of PCSA remains intact after fixing protocols necessary for preservation of biological samples and 4) laminin1 coating proved to be the most effective method for attaching neurons to the desired regions on PCSA discs. This work provides the basis for potential use of PCSA as a biomaterial scaffold for neural regeneration.

## Introduction

Aerogels are an attractive class of materials with tunable chemical, physical, and surface properties that have great potential for *in vitro* and *in vivo* applications. Aerogels were invented in the 1930's [1] and consist of a pearl-necklace-like network of nanoparticles leaving more than 90% of the bulk volume empty [2], [3]. While a variety of aerogel types have been synthesized [4], the core nanoparticles of most typical aerogels consist of silica. Of particular interest to the field of study presented in this work are the so-called crosslinked silica aerogels where significant mechanical strength has been imparted by crosslinking the silica nanoparticles covalently with polymers without significant compromise of the porosity and the low bulk density of the native material [5], [6]. The nano-porous, translucent, light-weight, and mechanically strong nature of crosslinked silica aerogels provides a unique set of features that renders this type of material an attractive candidate for a range of biomedical and biological applications from scaffolds and artificial membranes for cell growth and confinement, to medical inserts such as nerve repair conduits. Furthermore, the mesoporous nature of typical aerogels (pore diameters less than 300 nm) [5], [6] will allow exchange of nutrients and vital fluids without trapping the nerve cell body in the aerogel pores since most cell bodies are on the scale of several microns [7]. The low porosity and surface area of some commercially available membrane materials have been known to restrict cell growth. Aerogels on the other hand are materials with an extremely high surface area which makes them an attractive candidate for tissue growth applications which require large surface area to allow and encourage cell attachment [8].

Other advantages of exploring aerogels for *in vivo* applications are their excellent acoustic [9], [10], [11] and thermal [12], [13] insulating properties enabling external and non-invasive imaging techniques for aerogel-based implants, if positioned in the peripheral regions of the body. Also, the aerogel synthesis methodology is highly versatile, making it possible to create thick/thin aerogel coatings on other materials, self-supporting aerogel thin films and bulk aerogel-based structures. The aerogel surface can be made with varying degrees of surface wettability, which in turn offers an advantage over synthetic materials such as polydimethylsiloxane (PDMS), polylactic-co-glycolic acid (PLGA) and polycaprolactone (PCL) [14] that are naturally hydrophobic and require extensive surface modifications before these materials can be used for *in vivo* or *in vitro* applications. In general, aerogels provide a highly porous, three dimensional, and topographically rich plane. It has been proven that nanostructured topographical cues strongly influence cell adhesion, migration, and proliferation [15] making aerogels an attractive material for such studies. The three dimensional open pore structure of aerogels is particularly interesting since it mimics the three dimensional nature of tissue in organisms [16].

Previous studies conducted by the authors demonstrated the biocompatibility of polyurea-crosslinked silica aerogels by culturing proximal tubule-like opossum kidney (OK) cells in their presence for upwards of 10 days [17]. OK cell growth in culture or on the aerogels was comparable to their growth on tissue culture plastic. The biocompatibility of crosslinked silica aerogels has also been under investigation by Yin et al [18] demonstrating potential for use in blood implantable devices. Furthermore, the biocompatibility of silica particles has been widely explored and has shown a great deal of promise and compatibility with living matter [19], [20], [21], [22], [23], [24], [25], [26].

The present study was designed to evaluate the ability of sterilized polyurea crosslinked silica aerogels (PCSA) to support the growth of nerve cells in the absence and presence of the attachment-promoting materials: (1) poly-L-lysine (a synthetic polymer of L-lysine) which enhances attachment and growth of cells on culture surfaces due to its positive charge [27]; (2) basement membrane extract (BME), a complex mixture extracellular matrix proteins whose major components are laminin I, type IV collagen, entactin, and heparin sulfate proteoglycan [27], [28]; and (3) laminin1, which induces neurite outgrowth [29], [30], and promotes directional neuronal growth cone migration [29].

## Results and Discussion

**Figure 1:**
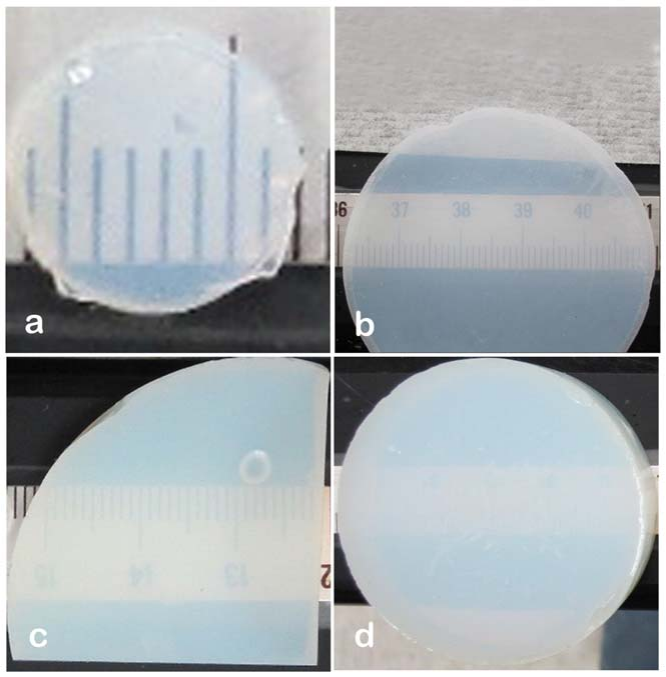
Aerogel translucency as a function of material thickness. Optical images of (a) 2 mm thick, (b) 3 mm thick, (c) 5 mm thick and (d) 10 mm thick PCSA discs with varying diameters, demonstrating the translucency of the PCSA discs as a function of thickness. All have densities close to 0.4 g/cm^3^.

Transparent and translucent biocompatible materials are advantageous over other material types since they permit microscopic observation of cells *in situ*. The nature of the study or application determines the thickness of the biomaterial needed requiring that (a) thin sections of the biomaterial should still have mechanical integrity; and, (b) the material's translucency/transparency should not be limited to thin sections only. For these reasons the translucency of polyurea-crosslinked silica aerogels was evaluated as a function of sample thickness. Discs with thicknesses of 2, 3, 5 and 10 mm and a density of 0.39 g/cm^3^ were cut from bulk to the desired thickness as shown in Figure 1 by means of a diamond saw. Due to the presence of the pores the visibility through the crosslinked aerogel decreases as material thickness increases. However, even for PCSA discs as thick as 10 mm the digits on the scale underneath the sample are still visible providing visual access to both sides of the membrane. In the case of discs with thicknesses 2 and 3 mm the PCSA has sufficient strength and material integrity that it can still be manipulated and processed extensively despite the thinness of the discs.

Sterilization is a key step in preparing a biomaterial and a simple UV/IPA treatment proved to be sufficient for this study. Static contact angle measurements before and after exposure to UV demonstrated that exposure to a flux of ∼2 W m^−2^ increased the surface hydrophilicity by almost 50%. The sessile drop test demonstrated a decrease in the receding angle from 61° to 31.20°. The increased surface hydrophilicity is expected to facilitate the adhesion of nerve cells and/or laminin1 to the aerogel surface.

**Figure 2:**
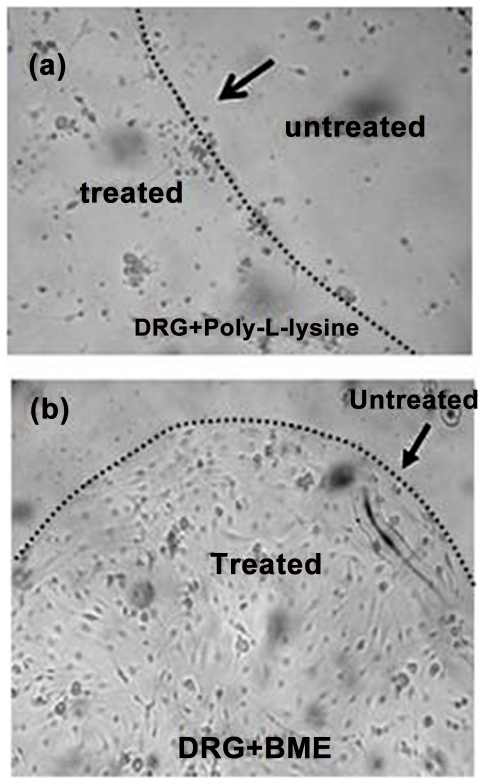
BME restricts nerve cell growth on glass. Optical microscope images of crystal violet stained with: (a) Poly-L-lysine coating (400× magnification) on glass coverslips not able to support growth and confinement of nerve cells.; (b) A 5 µl drop of BME (40× magnification) restricts nerve cell growth to the area of the drop. Arrows in both images indicating the boundary between the treated and untreated regions. Images were taken 5 days after plating.

**Figure 3:**
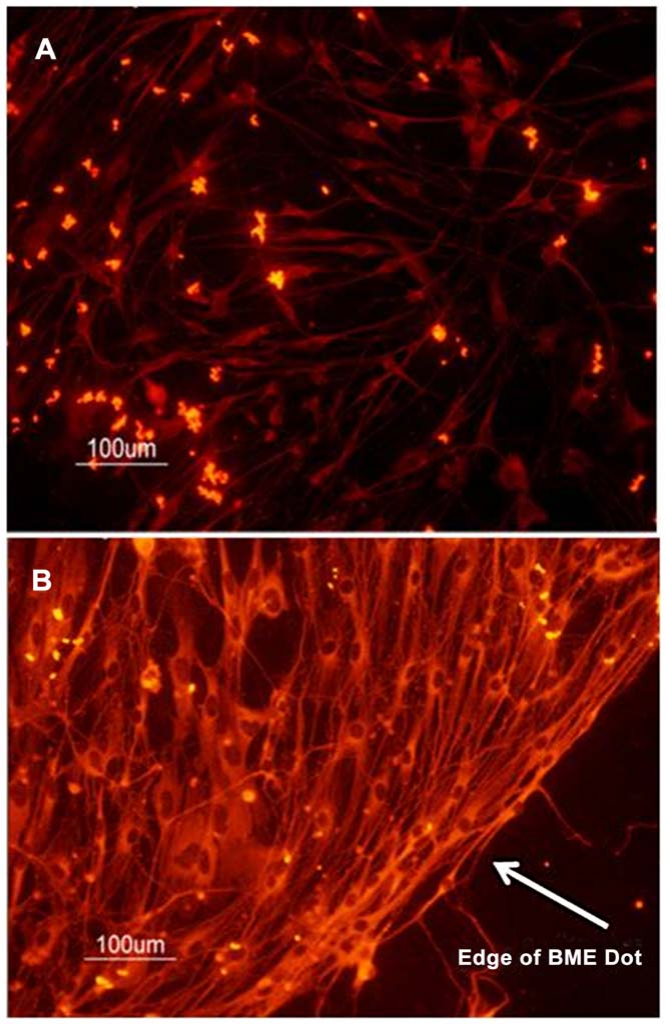
Immunofluorescent detection of nerve cells grown on laminin and BME. Fluorescent images of DRGs grown in 8 chambered glass slides in the presence of: (a) laminin; or, (b) BME. Nerve cells were visualized with an anti-bIII tubulin antibody. Arrow in (b) shows the edge of the BME dot. Images were taken fromcell fixed after 7 days in culture.

With the aid of crystal violet staining it was determined that poly-L-lysine did not promote attachment of DRG neurons to the glass slide (Fig. 2A). This likely reflects degradation of poly-L-lysine by proteases released by the DRG neurons [30]. In contrast, BME supports the confinement and growth of neurons onto glass (Figure 2B). In our next experiments, we assessed the ability of BME to promote neural cell growth and expression of the neuronal marker bIII tubulin while limiting the growth of non-neuronal cells likely to be present in the culture. Since BME is a complex mixture of extracellular matrix proteins including laminin1, we also evaluated the ability of laminin1 alone to promote attachment and neuronal differentiation. Immunofluorescence of the neural marker bIII tubulin of DRG neurons grown on laminin- and BME-patterned chambered slides showed that both laminin and BME promoted the growth of neurons without contaminating astrocytes as evidenced by the expression of bIII tubulin (Figure 3). Nerve cells grown on laminin had longer axons and fewer dendrites than did those grown on BME, consistent with the findings of Lein and Higgins [31]. Based on the fact that BME is a complex mixture of proteins and that laminin-cultured DRG neurons have a bipolar morphology more closely resembling that of DRG neurons *in vivo* we chose to use laminin1 as the attachment-promoting agent on aerogels.

**Figure 4:**
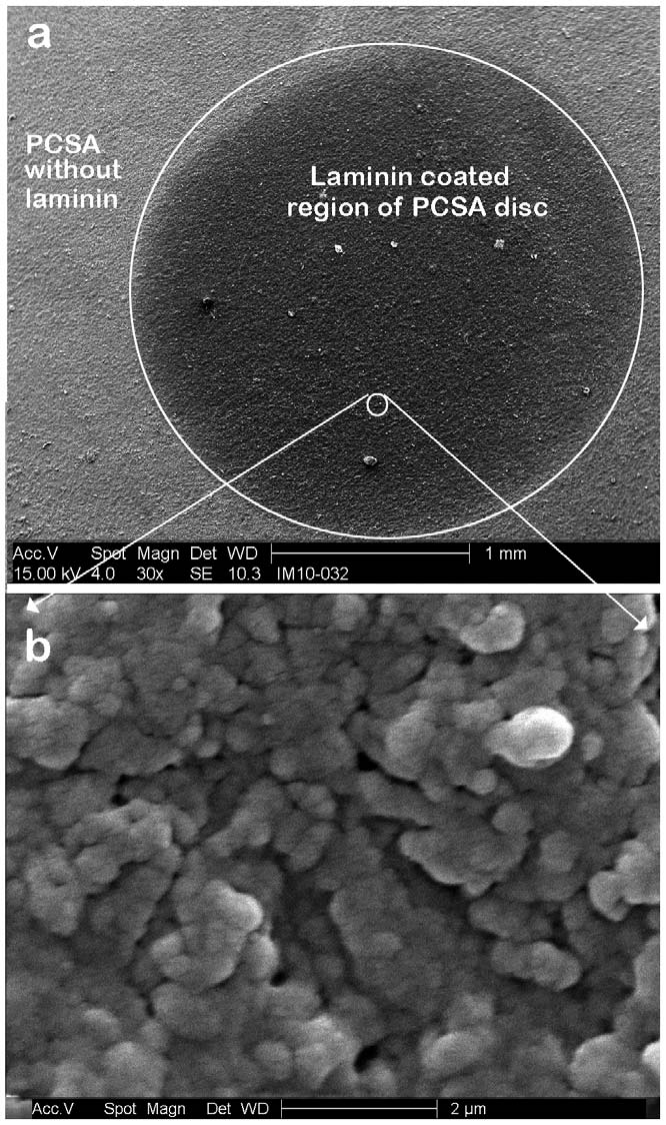
SEM image of laminin dot on PCSA. SEM image of: (a) 5 µl drop of laminin on a PCSA disc (post fixing/sputter coating stage). The dotted line shows the outline of the laminin coated region on the aerogel disc. (b) Higher magnification SEM image of the laminin coated region of the PCSA disc showing the aerogel pores with the laminin coating.

Figure 4 shows SEM images of a 5 µl laminin drop on the aerogel surface. The 5 µl drop covers a surface area of approximately 3.14 mm^2^ (Figure 4A). The extent of penetration of laminin into the pores of the aerogel cannot be judged accurately from the SEM images alone. A higher resolution SEM image (Figure 4B) shows that the three dimensional aerogel filaments sitting below the surface are indeed coated with the laminin and that the laminin layer does not block access to the pores.

**Figure 5:**
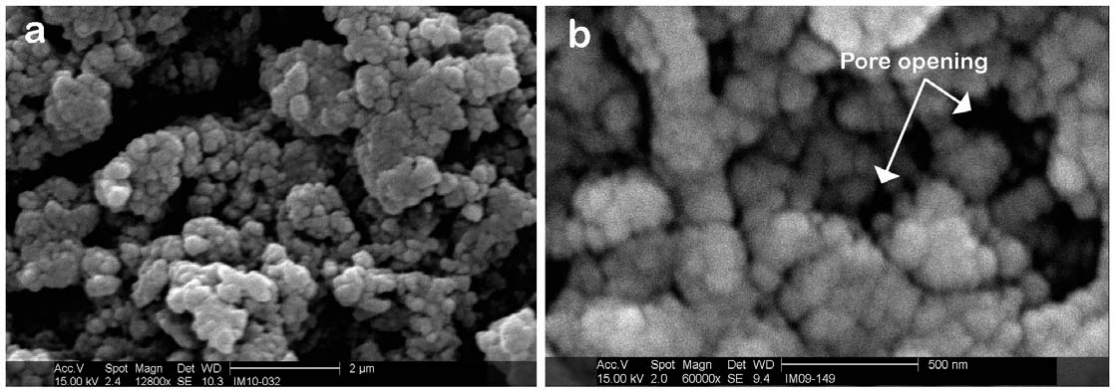
Aerogel pores intact after fixing treatment. (a) SEM image of PCSA after fixation and sputter-coating- off the laminin spot. The image shows the porous structure is still intact after the fixing treatment has been performed. (b) Image of a pore opening post fixing and sputtering stage.

Scanning electron microscope (SEM) images of the PCSA pores are shown after the fixing/sputter coating stage and without any laminin coating demonstrate that the aerogel pores are indeed intact and have not been perturbed by the fixing protocol (Figure 5). This is an important aspect of the aerogel as the porosity of a scaffold designed for nerve regeneration is necessary for vascularization and cell migration [14].

**Figure 6:**
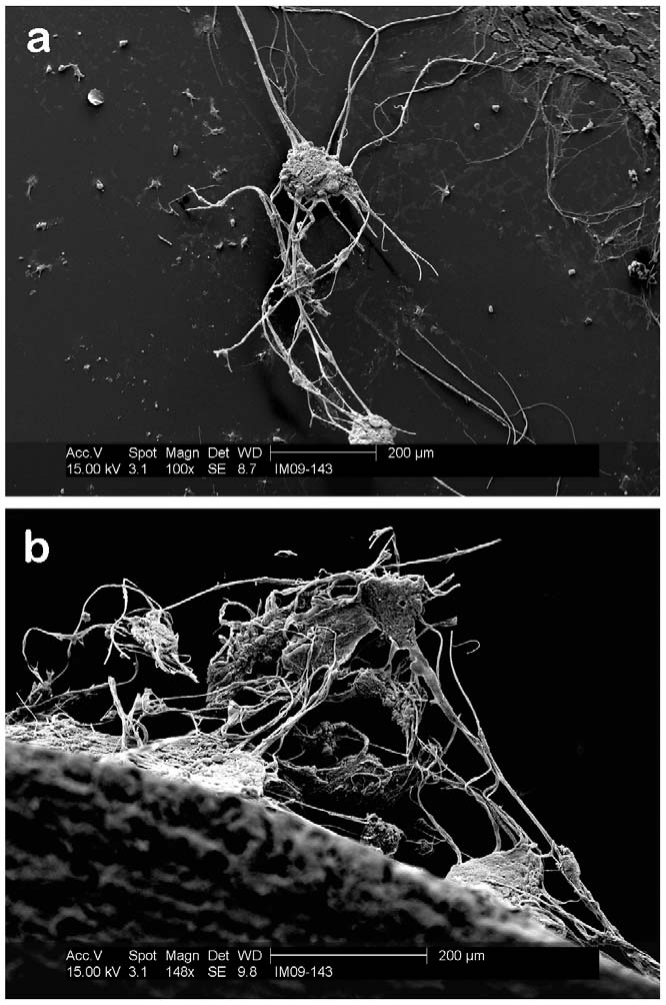
Neuron contact pattern. SEM images of neighboring neurons extending processes on: (a) surface of laminin-coated PCSA. Image was taken from the edge of PCSA disc capturing the interaction of several neurons. (b) Wide view of two neurons extending processes on a petri dish surface. Fewer processes have been extended in the latter case.

**Figure 7:**
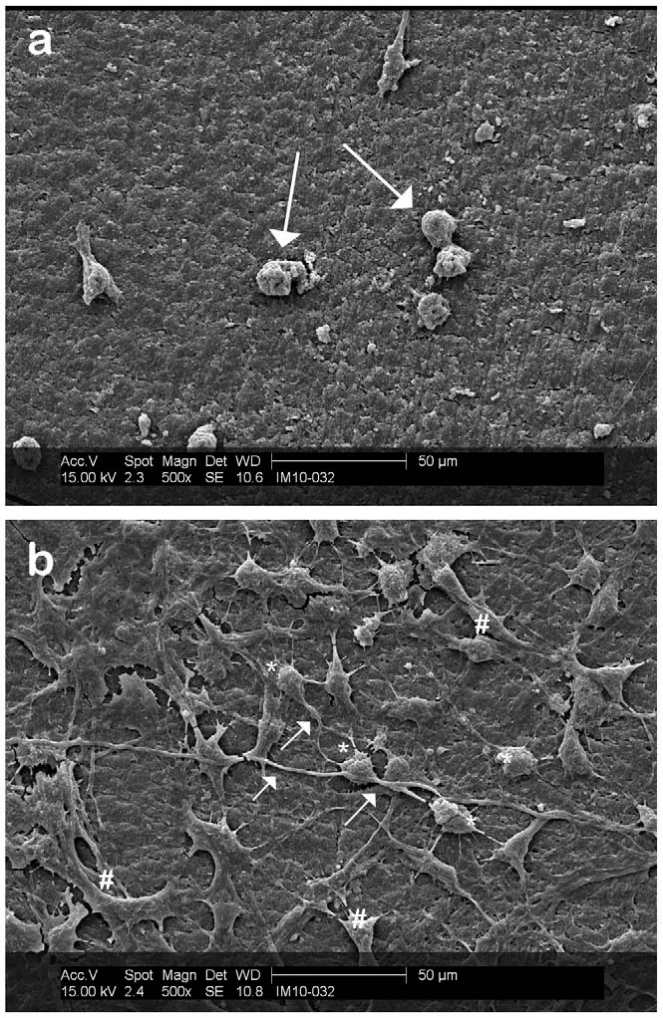
Effect of laminin on neuron growth. DRG neurons were plated on aerogel surfaces and grown for 7 days (to confluence) prior to processing for SEM. (a) SEM of nerve growth profile off the laminin dot, directly on the aerogel substrate. Arrows indicating attached nerve cells with no processes extended. (b) SEM of DRGs grown on a laminin drop on PCSA disc post-fixation and sputtering. DRGs cell bodies (*) are extending processes ( ) that interact with those of other nerves. Sample processing (freezing and sputter-coating) has lead to the fracturing of the lamimin layer (#). Images taken post-fixation and sputtering stage.

**Figure 8:**
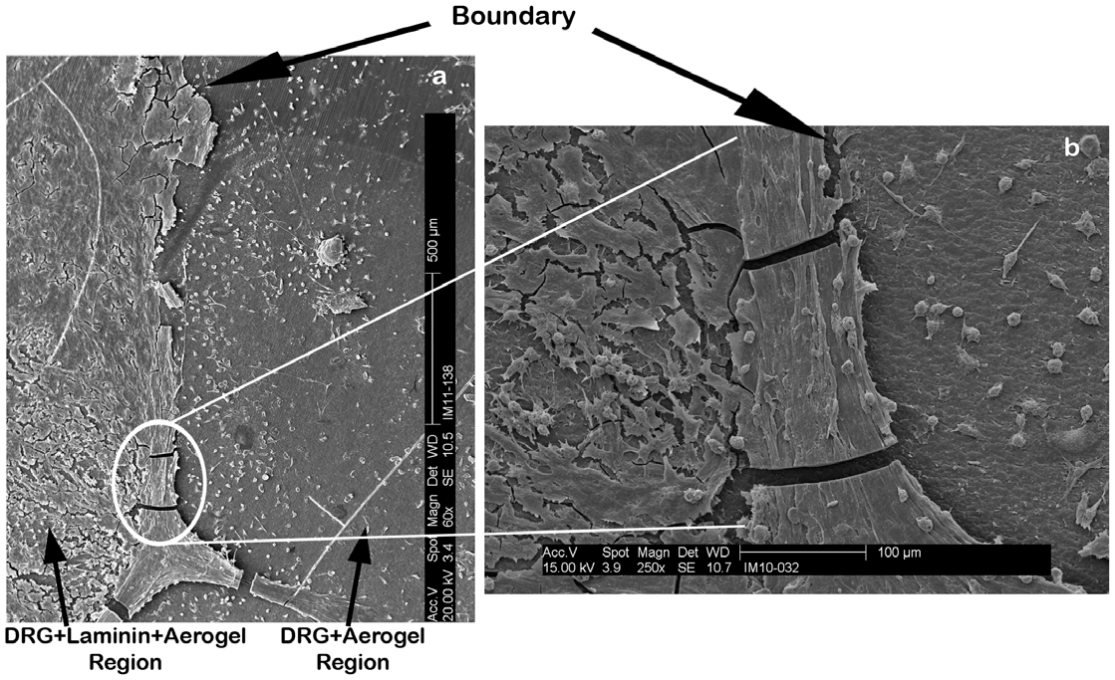
SEM images of laminin-aerogel-neuron interface on PCSA surface. (a) Growth of nerve cells on the laminin-coated section of the PCSA disc compared with growth of neurons directly on the aerogel surface in the absence of laminin (after 7 days). An abundance of cells have been immobilized directly on the aerogel surface but no processes have been extended. The laminin layer is necessary for processes to develop. (b) Higher magnification image of the boundary region highlighting the difference in the behaior of nerve cells on the different surfaces. Desnity of cells in both regions is equal. Dotted line shows boundary between the two regions.

The three dimensional interaction between DRG neurons on the laminin coated section of a PCSA disc is clearly captured in Figure 6A where a ganglionated group of cells growing on the surface and down the side of a laminin-spotted aerogel are imaged. The image was taken from the edge of the aerogel disc mounted on an SEM stub. It is clear that the laminin coated PCSA promotes the extension of processes and cell-to-cell interactions between DRG neurons. Figure 6B shows the behavior of DRG neurons on the tissue culture plastic well containing the aerogel with DRGs. DRG neurons are plated onto aerogels in 1 ml of medium and it is likely that some end up in the medium surrounding the aerogel. Comparing Figure 6A and 6B shows consistent pattern of neural networks established between neighboring DRG neurons. The significance of laminin coating on growth of DRG neurons on PCSA is further highlighted by comparing Figure 7A and 7B. Figure 7A shows attachment of some DRG neurons on the aerogel substrate but no processes have been extended from any of the cells. While the hydrophilic aerogel surface and the “grooved” surface morphology encourages the attachment, adhesion, and capture of some cell bodies, clearly, this is not adequate for promoting cellular interactions and is reminiscent of cells grown in the presence of poly-L-lysine (Fig. 2). Figure 7B however shows an abundance of processes and interactions between DRG neurons which is directly associated with the presence of the laminin layer as well as a higher density of cell bodies immobilized compared to Figure 7A. This point is emphasized when comparing the cellular behavior at the boundary of the laminin drop as shown in Figure 8. While cell bodies are present on the laminin free part of the aerogel surface, limited or no processes have been produced. From the SEM images it is clear that DRG neurons are not inclined to extend communication pathways on areas of the aerogel discs that have not been coated with laminin.

**Figure 9:**
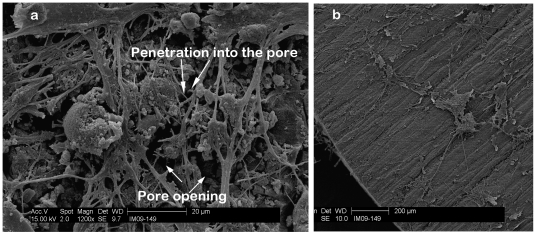
SEM image of neurons connecting on laminin-coated PCSA. (**a**) A dense array of processes have extended across the area of PCSA covered by laminin. Several layers of nerve cells with crisscrossing neuritis can be observed. The pores of the PCSA can be clearly seen and some processes may have extended into the pores as indicated by the arrows. (b) SEM image of the edge of a PCSA disc+Laminin+neurons showing the grooves created by the saw blade. It is hypothesized that the non homogoenetiy of the surface provides better anchoring and attachment oportunities for the cell body and the proceeses to be extended.

The laminin-coated region of the PCSA disc shows a dense layer of axonal growth and interactions between cell bodies at some distance from each other also reveals (Figure 9A) several layers of neurite and axons growing on top of each other. The opening of many pores can also be observed (two of which have been indicated with arrows) and some processes might have entered the pores.

In a recent review addressing the effects of three dimensional scaffolds on cell adhesion and growth, Li and Yang [16] indicated that surface geometry plays a role in adhesion as cells and fibroblasts prefer to attach to smooth surfaces while osteoblast-like cells attach to rough surfaces. Nerves also appear to have surface preferences as Long et. al [32] showed that micropatterned grooves on the inner surfaces of hollow fiber membranes promoted axonal outgrowth of DRG neurons. Thus, the wavy pattern created on the disc surfaces with the diamond saw during the slicing stage may enhance the adhesion of the DRG to the surface and provide better anchorage of the cell body with the aerogel surface (Figure 9B).

### Conclusion

By monitoring the interaction of dorsal root ganglia neurons on coated polyurea crosslinked silica aerogels we have demonstrated the ability of this material to promote the growth and differentiation of these cells. From the three coatings tested (poly-L-lysine, BME, and laminin), laminin proved to be the most effective surface treatment for attachment, confinement, and growth of DRG neurons on the PCSA surface. DRG neurons can attach to the untreated surface of PCSA but will not extend processes without a growth-promoting coating. The physical immobilization of DRG neurons on untreated PCSA is associated with the three dimensional and porous surface of the aerogels leading to attachment and immobilization of the cell body. Also, the porous nature of the aerogel remains intact after fixing treatment enabling further studies and image analysis. It is clear from the *in vitro* studies performed by the authors thus far that different cell types can be grown on PCSA membranes [17] and that the surface of PCSA is compatible with a variety of commonly-used popular coating materials. Therefore, PCSA shows promise as a synthetic scaffold supporting neuronal differentiation and future studies will evaluate its potential for the repair of nerve injuries.

## Materials and Methods

### 1 Synthesis of clear polyurea crosslinked silica aerogels (PCSA)

Two solutions, the first containing 3.85 mL tetramethoxysilane (TMOS) and the second one containing 4.5 mL methanol and 1.5 mL water as well as a 25 mL of 3-aminotrioxypropysilane were mixed in a 250 mL beaker with a sterile glass stirring rod. The resulting sol (colloidal suspension) was immediately poured into cylindrical molds and gelled within 60 sec while still cold. The gels were aged for 3 h in a methanol bath and subsequently washed with methanol (once) and four times with acetonitrile, using 4–5 times the volume of the gel for each wash. Subsequently, gels were transferred to an isocyanate bath containing 33 g of Desmodur N3200 (Bayer) in 94 mL of acetonitrile. The volume of the bath was again 4–5 times the volume of each gel. After 24 h, the gels were transferred to fresh acetonitrile and they were heated at 70°C for 72 h in a Blue-M Therm oven. At the end of the period, the gels were washed another four times with fresh acetonitrile (24 h each time) and then were dried by means of critical point drying, using liquid CO_2_ in a Polaron E3000. A Table top Buehler slow speed saw was used for cutting samples into thin discs of approximately 1 cm in diameter and 2 mm in thickness at half of its maximum speed.

### 2 Sterilization of aerogels

Aerogel discs were sterilized prior to cell plating and incubation by 1 h UV (254 nm) exposure - with a flux of ∼2 W m^−2^ on the top of the samples followed by a 5 min wash in 70% ethanol. The aerogel discs were then allowed to dry under the hood, in Pyrex petri dishes.

### 3 Surface contact angle measurements

In order to evaluate the effect of UV radiation exposure on polyurea crosslinked silica aerogel's surface conditions, the static contact angles were measured by means of a VCA Optima, AST Products, Inc contact angle measurement unit before and after the sterilization stage. A 2 µl drop of deionized (DI) water was deposited on the sample surfaces by means of a calibrated micro syringe. The receding and advancing angles were recorded using the Dynamic 2500 software.

### 4 Patterned growth of DRG neurons

To identify the extracellular matrix protein best supporting directed nerve cell growth, rat dorsal root ganglia (DRG) neurons (Lonza, Walkersville, MD) were cultured in eight chambered glass slides patterned with 1, 2.5 and 5 µl dots of poly-L-lysine (Thermo-Fisher, Pittsburgh, PA), laminin (Sigma-Aldrich, St. Louis, MO) and basement membrane extract (BME) (R&D Systems, Minneapolis, MN). Cells were visualized daily and in some instances, they were visualized by staining with crystal violet and photographed using a Canon 6 megapixel digital camera. As the DRG cultures may contain non-neuronal cells, the expression of the neuronal marker βIII tubulin was assessed by immunofluorescence. For this purpose, cells were grown for 5 days in 8 chambered glass slides patterned with either BME or laminin, then fixed in 4% formaldehyde, blocked with normal goat serum and incubated with a poly rabbit anti-βIII tubulin antibody (Milipore, Billerica, MA). Binding of the primary antibody was detected using a goat anti-rabbit IgG Alexa Fluor 488 antibody (Molecular Probes/Invitrogen, Carlsbad, CA) and visualized with a Nikon Eclipse E800 Confocal Microscope. After assessing neuron growth on patterned slides, BME and laminin were dotted onto sterilized PCSA discs 2 mm thick and 1 cm diameter. The BME or laminin dot coated the central region of the PCSA discs, leaving a boundary between coated and uncoated regions of the surface (Figures 2 and 4, respectively). Cell cultures on PCSA discs were visualized daily and the growth between coated and uncoated regions were also compared on a daily basis. PCSA discs were then evaluated by light and by scanning electron microscopy (SEM).

### 5 Preparation for SEM imaging

#### (a) PCSA+laminin+DRG fixing protocol

Of the different *in vitro* preparations discussed in section 2.4 only aerogel discs with the laminin coating were fixed and prepared for scanning electron microscopy. Three samples, (1) PCSA disc, (2) PCSA + laminin, and (3) PCSA+laminin+neuron were fixed in 2.5% glutaldehyde in 0.1 M sodium cacodylate buffer with a pH of 7.35 (Tousimis Research Corp) for 2 h. Samples were then rinsed for three times, 5 min each, with 0.1 M sodium cacodylate buffer at a pH of 7.35. Samples were post fixed with 2% osmium tetroxide pH 7.35 in 0.1 M sodium cacodylate buffer (Electron Microscopy Sciences) for 2 h. The samples were rinsed again for three times, 5 min each with 0.1 M sodium cacodylate buffer pH 7.35. Finally, PCSA discs were rinsed three times 5 min each rinse with DI water. Whole discs were stained with 2% aqueous uranyl acetate (Electron Microscopy Sciences) for 2 h. Discs were then rinsed 3 times, in 5 min durations with DI water. Samples were fully dehydrated through graded series treatment of ethyl alcohol (10% through absolute). Dehydrated samples were transferred to a Tousimis Sandai 790 Critical Point Dryer (CPD) in absolute ethyl alcohol. Critically point dried samples were then mounted onto SEM stubs.

#### (b) Sputter coating

Sixty nanometers of Au/Pd (60/40) (target purchased from Electron Microscopy Sciences (EMS), PA) was sputtered onto the samples prepared in section 4.5 by means of an EMS 550 sputter coater. A Philip XL 30 ESEM (Environmental Scanning Electron Microscope) was used for imaging of the fixed and sputter-coated samples.
